# A Digital Video and Text Messaging Intervention to Support People With Chronic Pain During Opioid Tapering: Content Development Using Co-design

**DOI:** 10.2196/40507

**Published:** 2022-11-10

**Authors:** Michael R Magee, Ali Gholamrezaei, Amy G McNeilage, Alison Sim, Leah Dwyer, Manuela L Ferreira, Beth D Darnall, Paul Glare, Claire E Ashton-James

**Affiliations:** 1 Pain Management Research Institute Faculty of Medicine and Health The University of Sydney Sydney Australia; 2 Consumer Advisory Group Pain Australia Deakin Australia; 3 Sydney Musculoskeletal Health, School of Health Sciences, Kolling Institute, Faculty of Medicine and Health, The University of Sydney Sydney Australia; 4 Department of Anaesthesiology, Perioperative and Pain Medicine, Stanford University School of Medicine Stanford, CA United States

**Keywords:** chronic pain, deprescribing, tapering, dose reduction, opioids, mHealth, mobile health, SMS, text messaging, digital health, behavior change, self-efficacy, consumer engagement, co-design, coproduction

## Abstract

**Background:**

People living with chronic pain report that tapering prescribed opioids is challenging and more support is needed. In our formative research, consumers indicated that mobile health (mHealth) technology could be an acceptable form of support for opioid tapering and may improve tapering self-efficacy.

**Objective:**

We aimed to evaluate and improve the content of an mHealth intervention before pilot-testing, based on consumer and clinician feedback.

**Methods:**

Participants were 12 consumers and 12 clinicians who evaluated an initial draft of a video script and 90 SMS text messages. Consumers and clinicians rated the appropriateness and likely usefulness (consumers) or likely effectiveness (clinicians) of a video script and a random selection of 15 SMS text messages using a 5-point Likert-type scale (1=totally disagree; 5=totally agree). Each draft SMS text message was reviewed by 2 consumers and 2 clinicians. Texts were deemed acceptable for inclusion in the pilot intervention only if the summed participant ratings of text appropriateness and usefulness or effectiveness were ≥8. Participants were also invited to provide open-text feedback on the draft script and SMS text messages.

**Results:**

Consumers generally agreed that the draft video script and text content were likely to be appropriate (video: mean 4.4, SD 0.52; text: mean 4.3, SD 0.79) and useful (video: mean 4.3, SD 0.65; text: mean 4.2, SD 0.84). Similarly, clinicians generally agreed that the draft video script and text content were likely to be appropriate (video: mean 4.5, SD 0.67; text: mean 4.4, SD 0.81) and effective (video: mean 4.0, SD 0.43; text: mean 4.3, SD 0.76). Overall, 77% (69/90) of the draft texts met the threshold rating for acceptability for inclusion in the pilot test of mHealth intervention by consumers, and 82% (74/90) met the threshold for acceptability by clinicians. Consumers' and clinicians’ ratings were used to rank order the texts. The top 56 draft texts (all meeting the threshold levels of acceptability) were selected for inclusion in the pilot intervention. When consumer or clinician feedback was provided, the texts meeting the criteria for inclusion in the pilot were further revised and improved. Feedback on the video script was also used to further improve the acceptability of the video script before pilot-testing the intervention.

**Conclusions:**

This study describes the process by which a 28-day mHealth intervention to support patients with chronic pain to taper opioid medications was evaluated and improved before pilot-testing. The mHealth intervention consisted of a 10-minute psychoeducational video about pain and opioid tapering and 56 unique SMS text messages providing information and reassurance (texts delivered twice per day for 28 days). Having established that the content of the mHealth intervention is acceptable to both consumer and clinician groups, the mHealth intervention will be piloted in future research.

## Introduction

### Chronic Pain and Opioid Tapering

The reduction or cessation of prescription opioid medications (opioid tapering) is recommended for people living with chronic nonmalignant pain in cases where the potential harms of long-term high-dose opioid therapy outweigh the benefits [1-4]. Tapering can lead to unpleasant withdrawal symptoms, increased distress, and a potential increase in pain intensity and interference, especially in the absence of alternative nonpharmacological pain management strategies [3-7]. Research suggests that consumers with chronic pain, who are tapering opioids, benefit from education regarding the negative consequences of long-term opioid therapy, nonpharmacological (ie, cognitive and behavioral) active pain management strategies, and strategies to help with the management of withdrawal symptoms [1,8]. Consistent with this, research indicates that patients with chronic pain who have access to multidisciplinary pain management programs (with care provided by a team of physicians, physiotherapists, and clinical psychologists) are more likely to reduce their opioid dose with fewer negative side effects [8,9]. However, there is a limited workforce of physicians, psychologists, and physiotherapists with expertise in chronic pain treatment, which is far outweighed by consumer demand for this specialized support [10-12]. Hence, there is a need for innovative, scalable solutions to provide specific support to people living with chronic pain who are tapering opioids [10,13].

### Digital Health

Digital and mobile health (mHealth) services might help address this gap in access to support for opioid tapering in patients with chronic pain [13,14]. Accumulating research indicates that mHealth interventions may be an acceptable form of opioid tapering support for individuals living with chronic pain [15,16]. SMS text message–based interventions, in particular, may be most effective in reaching the largest number of consumers, as texts do not require access to the internet, have low technology literacy requirements, and are low cost and familiar, as there is a high current use of text messaging worldwide [16-18]. Mobile phone text interventions have demonstrated effectiveness in facilitating health-related behavior change [18,19], helping people to quit smoking [20], improving outcomes in the management of musculoskeletal conditions [21], and even supporting people to manage perioperative pain [17,22].

### Co-design

Although text message–delivered mHealth interventions have the potential to be efficacious, the effectiveness of digital health interventions in practice is likely to be enhanced by consumer co-design. Research demonstrates that using a co-design methodology in the development and evaluation of mHealth interventions improves their acceptability, increases the likelihood that researchers will achieve recruitment and retention goals in clinical research trials, and ensures that interventions meet the needs of targeted populations, such as people living with health issues like chronic pain [23-27]. Our research group used a 4-step co-design methodology [28] to develop and test an effective mHealth intervention to support patients with chronic pain to taper prescription opioids. The co-design steps include (1) conducting formative research with consumers to develop insights into consumer needs and preferences, (2) developing the content of the intervention to meet consumer-stated needs and preferences, (3) coevaluating the intervention content in consultation with consumers and key stakeholders (eg, clinicians or caregivers), and (4) revising and improving the intervention in response to feedback [28].

Previously, we conducted formative research with consumers to identify whether people living with chronic pain who were tapering their opioid medication dose reported favorable attitudes toward the feasibility and acceptability of mHealth interventions to support tapering [16]. Our formative research revealed the following key insights:

1. For most consumer participants, SMS text messaging was preferred over an app for the delivery of support [16].
2. Consumers wanted the mHealth intervention to provide emotional and informational support concerning managing chronic pain without opioids; withdrawal symptom management; and strategies for improving functioning, quality of life, and mood [16].
3. Consumers suggested that text message–delivered support would be more engaging and effective if they had some familiarity with the information being provided (ie, pain self-management strategies) [16].

On the basis of this consumer feedback, and in collaboration with a consumer with lived experience (LD), as well as with clinician representatives (AS, BDD, MLF, MRM, and PG), we developed the concept of a text-delivered mHealth intervention to support patients with chronic pain to taper prescription opioids. The content of the intervention should be developed to provide consumers with emotional and informational support with respect to pain self-management and strategies for minimizing withdrawal symptoms and improving mood. In response to consumers’ suggestions to familiarize users with intervention content before it is received through texts, we identified the need for a brief preintervention psychoeducational video. Supporting the benefits of including an educational video in the mHealth intervention, research indicates that after viewing a brief educational video, including patient testimonials about the benefits of opioid tapering, patients develop more positive attitudes toward tapering [29]. Further research indicates that videos including testimonials about the effectiveness of digital health interventions can improve their acceptability [30].

### Self-efficacy and Behavior Change

Consumers are more likely to engage with and respond to health interventions rooted in behavior change theory compared with interventions that are not theory-derived [31,32]. Research investigating the experiences of people who are tapering opioids suggests that low self-efficacy is a barrier to tapering and that improvements in both pain self-efficacy and opioid tapering self-efficacy are associated with improved outcomes for people living with chronic pain who are tapering [15,33]. Self-efficacy for opioid tapering has been demonstrated to be amenable to change through digitally delivered education [29,34,35]. Self-efficacy, a concept similar to confidence, refers to an individual’s self-appraisal of their ability to perform a certain task or achieve a specific outcome [34,35]. Social learning theory suggests that enhancing self-efficacy for specific health-related behaviors is one of the key drivers for enduring behavioral change [36]. In 1 study that delivered a digital pain self-management program, improved pain self-efficacy was associated with reduced opioid misuse [37]. Accordingly, we theorized that self-efficacy to taper should be associated with a greater likelihood of reducing reliance on opioid medications to manage pain. As such, improving opioid tapering self-efficacy was posited as the proximal goal of our mHealth intervention [28].

### This Study

Building on our formative research, this study describes the process by which mHealth intervention content was developed to meet consumer-stated needs and preferences, evaluated in consultation with consumers and key stakeholders (clinicians), and revised in response to feedback.

## Methods

### Setting

The study was conducted within a specialist pain clinic and research institute at a metropolitan public hospital in Sydney from December 2020 to August 2021.

### Participants

Purposeful recruitment was selected as our aim was to develop an intervention for a very specific population: people with chronic pain who were tapering their opioid medications under the supervision and guidance of a pain specialist [38]. Hence, for the initial evaluation of intervention content, we recruited consumers from specialist pain clinics and clinicians with experience in delivering tertiary care to patients with chronic pain who are tapering opioids. Within this specialized consumer population, we aimed to recruit a diverse sample of participants regarding socioeconomic, cultural, gender, linguistic, and other characteristics. We drew on our extensive Australia-wide network of clinical colleagues to identify patients with chronic pain who had some experience of tapering opioids (not necessarily successful or good experiences).

Overall, 12 individuals with chronic pain who had previous experience tapering prescription opioid medications were recruited through purposeful sampling and targeted recruitment [38]. The 12 consumers varied with respect to their education, income, culture, preferred language, and geographic region (metropolitan, regional, and rural). These consumers who participated in the evaluation of video and SMS content had varied tapering experiences. Most consumer participants had experienced difficulties with tapering. Some had been successful in overcoming a reliance on opioids to reduce their dose. Other participants were still tapering or had paused their dose reduction. Hence, our consumer participants had experience of what does not work, what is hard about tapering, and what can be helpful. They were known to the researchers as consumers of pain services through previous research participation and consumer involvement, affiliation with an Australian consumer advisory group [39] or had previously expressed interest in pain research or consumer advocacy. Of these 12 consumers, 4 (33%) were born overseas, while 8 (67%) were born in Australia. Three participants lived in regional areas, and the remainder lived in metropolitan areas.

We matched our sample of consumers with 12 clinicians from varied pain management disciplines. The sample included registered multidisciplinary health professionals who currently work with patients who experience chronic pain and have also supported consumers to reduce or cease opioid medications directly (ie, the physician supervising dose reduction) or within a multidisciplinary team (eg, a psychologist or physiotherapist in an individual or group pain program supporting consumers who are tapering). The 12 clinicians included 5 specialist pain physicians (3 anesthetists, 1 neurologist, and 1 psychiatrist), 3 clinical psychologists, 3 physiotherapists, and 1 osteopath, who were practicing in private and public clinics in rural or regional and metropolitan clinical settings. Clinicians were recruited through purposeful sampling and direct invitation. The clinicians invited to participate in this study all delivered evidence-based interventions within pain management programs, supported consumers who were tapering prescription opioids, had diverse clinical experiences across different areas of Australia (ie, working in private and public clinics, working across regional and metropolitan areas, and supporting consumers with diverse cultural and linguistic backgrounds), and kept up to date with research and treatment advances in the area.

### Ethical Considerations

This study was approved by the Northern Sydney Local Health District Research and Ethics Committee (2020ETH03288). All participants were provided with study information before completing the surveys. As indicated in the study, participants provided informed consent. To ensure privacy and confidentiality, only minimal demographic information was collected from participants. No compensation was provided for participating in the study.

### Initial Design Considerations for the Content of the mHealth Program

#### Overview

In response to consumer feedback, the mHealth content design and aims are to support users to successfully reduce or discontinue their prescription opioid medication when engaging in a voluntary taper. On the basis of self-efficacy theory [34,36], as well as clinical resources and research on pain management [40-46] and opioid tapering [6,33,37,47-50], members of the research group (MRM, AG, AGM, CEAJ, and PG) drafted content for the preliminary video script and texts. This content was drawn from digital and traditional written resources and included information about pain self-management education, cognitive behavioral therapy concepts, and informational and supportive content from previous chronic pain and tapering research [7,9,15,16,40,42-45,47-51]. The researchers who developed the draft script and texts screened and adapted the information and resources to develop content that matched consumer-identified themes from formative research [29]. The other authors (AS, LD, BDD, and MLF) provided iterative feedback on the draft content and supported further editing of the script or texts.

#### Video Design

The video was designed to familiarize participants with information about chronic pain, provide a rationale for opioid tapering (eg, emphasizing long-term side effects and enhancing motivation to taper [29,30]), strengthen opioid tapering concepts (benefits of tapering on quality of life and functioning), and address common concerns about tapering (withdrawal management and information on pain intensity) [6,33,37,47-50], while introducing various pain self-management strategies (including flare-up management, relaxation, sleep hygiene, thought management, and pacing) [40-46]. These psychological concepts were also linked to improving mood, and the association of tapering, mood, and pain was also included in the content [15,42,43,46,52]. To reinforce informational content, 3 patient testimonials were solicited and included in the video design. Previous research has demonstrated that patients who watch testimonials express more favorable attitudes toward opioid tapering than those who receive information without testimonials [29]. On the basis of the informational content, a series of slides with accompanying images were developed by members of the research team (MRM, AG, AGM, CEAJ, and AS). The video script, including testimonials, was transcribed for review. First, by the rest of the authors, then following further revisions, the script was prepared for review by consumers and clinicians for this study.

#### Text Message Design

The texts aimed to provide daily informational and emotional support and reinforce key concepts about pain management and opioid tapering, particularly those concepts introduced in the video. The research groups (MRM, AG, AGM, CEAJ, and PG) generated 200 texts in the first instance. The team (all authors) made initial revisions. Duplicates were removed and the texts were edited for an appropriate length; 160 characters or less was optimal, but texts of up to 320 characters (2 standard texts) were permitted. The language of the texts was also simplified throughout revisions. Following these edits, the list was reduced to 90 texts. The texts included a range of content based on the communication objectives, information, and resources cited earlier. Content addressed pain education and self-management strategies and information about opioids and opioid tapering. Texts were designed to enhance motivation to taper opioids, provide emotional support (ie, messages were validating and encouraging), minimize nocebo, and optimize placebo. Content was also designed to be engaging, principally because engagement with mHealth content may improve the likelihood that users achieve their behavioral change goal [53-56]. Specific strategies to increase engagement included simplifying the reading load and including validating [57], reassuring [58], encouraging, motivating [59], and supportive content [60,61]. Texts were also designed to be personalized, with intermittent use of the users’ preferred name [18,61,62]. Content was tailored for specific times of the day [19]. For example, sleep hygiene information was included in evening texts and motivational content was included in morning texts [61].

### Sample Size

It was determined that 12 consumers would be needed to ensure that all messages could be evaluated by 2 participants from each group. The sample size was estimated based on similar previous designs, considering that to obtain sufficient qualitative and survey data to evaluate a message, each total SMS text message should be reviewed by at least 2 clinicians and 2 consumers [41]. However, to reduce the burden and in the hope that more content would be deeply reviewed, we wanted only 15 messages reviewed per participant. Overall, 12 participants per group (consumers and clinicians) provided 2 opinions from each group for every 15 messages.

### Stakeholder Evaluation of Intervention Content

Consistent with previous research [28,40], the proposed content of the mHealth intervention was next evaluated by patients with pain who had experienced tapering opioids (consumers) and clinicians with experience supporting patients with pain to taper opioid medications.

### Procedure

Consumers and clinicians who consented to participate were sent a web-based survey using REDCap (Research Electronic Data Capture; Vanderbilt University) [63]. The participants were asked to provide feedback on the video script and 15 texts from the text library. Each text was reviewed by 2 consumers and 2 clinicians.

### Measures

Consumers were asked to rate the appropriateness (“easy to understand, sensitive to individual circumstances, and accurate”) and usefulness (“supporting and motivating patients to persist with reducing their dose of opioids”) of the script and SMS text messages on a 5-point Likert-type scale (1=totally disagree, 2=disagree, 3=neutral, 4=agree, and 5=totally agree). Clinicians rated the appropriateness and effectiveness (“supporting and motivating patients to persist with opioid tapering”) of the script and SMS text messages on the same scale. All participants were invited to provide open-text feedback about the script and SMS text messages. This methodology was adopted from a similar study that co-designed texts for an mHealth intervention [41].

### Analytic Strategy

For the video script, the agreed appropriateness, usefulness, and effectiveness were evaluated by reviewing all participant responses to calculate the mean ratings of the appropriateness (both groups), likely usefulness (consumers only), and likely effectiveness (clinicians only) items. It was predetermined that the average appropriateness, usefulness, and effectiveness scores of “agree” (mean 4) or “totally agree” (mean 5) would indicate the acceptability of the script. Open-text feedback was also reviewed by the research group to improve the script and texts.

For the texts, as with the script, the average appropriateness, usefulness, and effectiveness scores of “agree” (mean 4) or “totally agree” (mean 5) would indicate the overall acceptability of the content. Unlike the script, in which all participants reviewed the same content, each text was evaluated by only 2 consumers and 2 clinicians. This was to make it more feasible for the study participants to complete the survey. The mean sum score (MSS) was calculated and used to rank each text. For each text, the consumer MSS is the sum of the appropriateness and usefulness ratings for the 2 consumers divided by 4. For each text, the clinician MSS is the sum of the appropriateness and perceived effectiveness ratings for the 2 clinicians divided by 4. The MSS has a range of 0 to 10. The objective of this analysis was to identify the most suitable messages for inclusion in mHealth support. Suitability was determined as an MSS that would suggest most responses on the usefulness and effectiveness scores were “agree” (mean 4) or “totally agree” (mean 5). An MSS <8 for clinicians and consumers was set as a cut-off for inclusion in the final mHealth content [41]. Open-text feedback was also reviewed for each text message and used to guide the revision of text messages. MSS was used to rank each text message for revision and inclusion decisions, which are described in the Results section.

## Results

### Overview

The Multimedia Appendix 1 contains a detailed breakdown of descriptive statistics for survey items (scripts and texts) and a summary of the wide range of open-text feedback toward the content provided by consumers and clinicians. Key results are also described in subsequent sections, generally separated by script or texts and consumers or clinicians. Descriptive statistics for the script and text survey results (both groups) were not normally distributed, so median and IQR values were reported.

### Video Script

Table 1 reports the survey results for consumer and clinician ratings of the video script. On average, consumers endorsed responses indicating they “agreed” the video script was appropriate and likely to be useful. On average, clinicians endorsed responses indicating they “agreed” the video script was appropriate and likely to be effective. In total, 83% (10/12) of consumers provided feedback on the script. The video script was praised for the broad range of pain self-management strategies provided (“[It] gives a full snapshot”) and for the inclusion of testimonials (“Seeing someone that has gone through something similar will be beneficial”). Other suggestions were used to revise the video script (described here), such as providing more *“*coping mechanisms,*”* emphasizing the “real and tangible” benefits of tapering and clarifying the difference between acute and chronic pain. Overall, 58% (7/12) of clinicians provided qualitative feedback on the script. The video script was praised for the use of simplified language (“Obviously a lot of thought has gone into the lay examples and terms”), the repetition of “values-based activities,” and the use of real patient testimonies (“The cases can be very helpful”). Other suggestions were used to revise the video script (described here), such as changing the metaphor used to describe chronic pain (“I find people relate more to a faulty smoke alarm rather than a car alarm”) and providing more validation (“I wonder if less information and more recognition of potential emotional distress might be helpful”).

**Table 1:** Descriptive statistics for script.

Question			Value, mean (SD)		Value, median (IQR)		Range (minimum-maximum)
**Appropriate^a^**							
	Consumer	4.42 (0.515)		4 (1)		1 (4-5)	
	Clinician	4.50 (0.674)		5 (1)		2 (3-5)	
**Likely useful^b^**							
	Consumer	4.33 (0.651)		4 (1)		2 (3-5)	
**Likely** **effective^c^**							
	Clinician	4.00 (0.426)		4 (1)		2 (3-5)	

^a^Appropriate: easy to understand, sensitive to individual circumstances, and accurate.

^b^Likely to be useful: supporting and motivating patients to persist by reducing their dose of opioids.

^c^Likely to be effective: supporting and motivating patients to persist with opioid tapering.

### Text Messages

Table 2 presents the descriptive statistics of the text message survey. On average, consumers indicated they “agreed” the texts were appropriate and likely to be useful. On average, clinicians indicated they “agreed” texts were appropriate and likely to be effective. The mean of all text sum scores for both consumers and clinicians was >8 (acceptability cut-off for texts). A total of 77% (69/90) of texts had a consumer MSS ≥8, and 82% (74/90) of texts had a clinician MSS ≥8. Of the 90 total texts, 41 (46%) had consumer comments and 28 (31%) were commented on by clinicians. Overall, 10 consumers and 8 clinicians provided general open-text feedback on the text content. They made suggestions about the frequency (eg*,* “Every day is great,” consumer; “I think two per day,” clinician), timing (eg, “First thing in the morning for motivation and in the afternoon when we start lagging,” consumer; “One to start the day that has some strategies e.g. relaxation and one at the end of the day/late arvo that is more reassuring,” clinician), and tone (eg, “Change the language to a more active voice rather than passive,” consumer; “Less bossy,” clinician) of the text messages. Over 80% of clinician and consumer responses to individual texts endorsed participants “agreed” (mean 4) or “totally agreed” (mean 5) the texts were appropriate, likely to be useful, and likely to be effective. There was considerable agreement between groups. Overall, 66% (60/90) of the total texts were rated MSS <8 by both groups. In total, 10% (9/90) of texts were rated MSS <8 by consumers but MSS <8 by clinicians; 16% (14/90) of texts were rated MSS <8 by clinicians, but not by consumers. Overall, 8% (7/90) of texts were rated MSS <8 by both groups.

**Table 2:** Descriptive statistics for text message survey.

Question		Value, mean (SD)	Value, median (IQR)	Range (minimum-maximum)
**Appropriate^a^**				
	Consumer	4.26 (0.785)	4 (1)	3 (2-5)
	Clinician	4.38 (0.807)	5 (1)	4 (1-5)
**Likely useful^b^**				
	Consumer	4.23 (0.840)	4 (1)	3 (2-5)
**Likely effective^c^**				
	Clinician	4.26 (0.758)	4 (1)	4 (1-5)
**Sum score^d^**				
	Consumer	8.49 (1.537)	8 (2)	6 (4-10)
	Clinician	8.55 (1.291)	8 (2)	6 (4-10)

^a^Appropriate: easy to understand, sensitive to individual circumstances, and accurate.

^b^Useful: supporting and motivating patients to persist with reducing their dose of opioids.

^c^Likely to be effective: supporting and motivating patients to persist with opioid tapering.

^d^Sum score (both survey items for texts combined for each participant).

### Revise mHealth Intervention

#### Video Script

On the basis of consumer and clinician feedback (see Multimedia Appendix 1 for a full summary), the following revisions were made to the draft video script. The overall length was shortened because of feedback that “the script is long. It takes many paragraphs to get to the point that the study is about supporting opioid reduction because opioids don’t ‘work’ for chronic pain.” The script initially described both chronic and acute pain in detail, with comprehensive definitions of both. The content in the revised script introduction was narrowed in length and focus to provide mostly an introduction to, and education about, chronic pain. A metaphor used to explain chronic pain was changed as suggested by a clinician. Emphasis on pain self-management strategies was prioritized. Information about the mHealth intervention was reduced, with a focus on increasing positive expectation bias, nocebo effects, and acceptability, rather than explaining the procedure of the study. On the basis of feedback, acknowledgments and normalization of tapering concerns were added to the introduction, and reasons for tapering opioids (ie, the benefits of reducing dose and the harms of continued use) were emphasized. The language used in the video was further simplified and changes were made (broadly and based on specific feedback). For example, clinicians suggested that the term “tapering” could be simplified to “reducing your dose,” and this change was made in both the script and most texts. Finally, we added to the script to emphasize the importance of working collaboratively with the GP or prescribing physician.

Suggestions were made to change the content of consumer testimonials. As these testimonials had already taken place and the content could not be changed, the research group conducted interviews with 2 additional consumers (including culturally and linguistically diverse participants). The additional content included more discussions of strategies that were found helpful during tapering (eg, pain flare management). The interviewees also discussed their perspectives on managing chronic pain and the benefits of tapering opioid medications. Interview videos were edited using Adobe Premier Pro [64] video editing software, and informational content was recorded using Microsoft PowerPoint [65]. The slideshow and consumer videos were then iteratively edited, improving the quality of the audio recording and making changes to images used in the slide deck. Finally, the video was uploaded to a web-based video streaming platform so that it could easily be made available for viewing within an internet browser. A transcription was prepared for closed captions for those with hearing or comprehension difficulties. As the video contains recordings of consumers, it is not publicly shared; however, a full transcript of the final script is included in Multimedia Appendix 1.

#### Text Messages

The texts were edited based on survey responses and open-text feedback. All texts were placed in rank order using their MSS score. First, the texts were ranked by consumer MSS. If the consumer MSS was the same, those scores were ranked by the clinician MSS. All individual text feedback was reviewed by members of the research team, even those with an MSS ≤8 in either group (ie, those that were not considered for inclusion in the final list). Where there was a large divergence between group MSS, or there was conflict in the open-text feedback, revisions attempted to address the concerns but not in a way that interfered with the original intention of the text (ie, the communication strategy). Texts were not included in the final list if the MSS for that item (across both clinicians and consumers) was ≤8. For each text, based on the evaluation score and feedback, it was decided to keep, modify, or remove the messages. Of the 90 messages, 57 (63%) were commented on, 36 (40%) modifications were made, and 3 (3%) texts were removed from the draft message bank. These comments also resulted in the development of 2 additional messages. The 2 examples are listed in Table 3. The final message list and further examples of revisions are provided in the Multimedia Appendix 1.

**Table 3:** Examples of text revisions.

Original message	Feedback	Decision and revised message
Message 7, pain education and self-management: “Relaxation strategies can help to reduce muscle tension and pain. Try deep breathing exercise for a few minutes several times per day.”	Consumer MSS^a^ 9; consumer feedback: “Link with doing relaxation with somebody if really tense or use a tape that is specific to you.” Clinician MSS 9.5; no clinician feedback.	Revised message, included in the final list: “Relaxation strategies can help to reduce muscle tension and pain. Try deep breathing exercise for a few minutes several times per day. Finding a recording online that works for you can be a great help too! USYD”
Message 35, opioid tapering support: “Opioid medications can be useful for pain in the short term (a few days or weeks). However, research shows that opioids offer only limited relief in the long term.”	Consumer MSS 7.5; consumer feedback: “It is important to remember that if you have had experience with long-term opioid use, even short-term use will need to be closely monitored as it can open you to thinking you may need them long term again.” Clinician MSS 6; clinician feedback: “It seems like a backward step to receive a message promoting opioids for acute pain and irrelevant.”	Removed message from the library. Accept feedback from the clinician reviewer. Unnecessary message.

^a^MSS: mean sum score.

In response to general open-text feedback on the texts, the decision was made to include 2 texts per day, with 1 text in the morning and 1 in the afternoon for 28 days. The morning texts were sent between 9 AM and 11 AM, and the afternoon texts were sent between 2 PM and 5 PM. The texts were reviewed to ensure that the communication strategy was effective throughout the day. Texts were also reviewed to ensure that the language was simple, that the key concepts were concisely delivered, that language was persuasive without being “bossy” (a specific piece of feedback), and that an active voice was used for more of the texts. The sign-off “USYD” (for the University of Sydney) was added to the end of each text to ensure consistency and clarity for consumers [40].

In total, 54 of the draft texts were included in the final text list. In total, 59% (32/54) of the texts in the final list received qualitative feedback from a consumer or clinician, and 19% (10/54) were modified based on feedback during the review. Of the 54 final texts, the MSS for consumers was 8.98 (SD 0.693), and the MSS for clinicians was 8.981 (SD 0.725). Of 32 “pain education and self-management” texts, 17 (53%) were in the final list. Of 32 “motivation to taper opioids” texts, 19 (59%) were in the final list. Of 26 “opioid tapering support” texts, 18 (69%) were in the final list. The remaining 2 texts included an introductory text on commencing the study (day 1, text 1), which asked participants to “Save this phone number as TXTSupport@USYD” and another text that included links to web-based pain management resources (day 1, text 2). One “motivation to taper opioids” message was modified to include a final message for participants (day 28, text 2). To ensure fidelity for the evaluation of the content, texts were standardized so that the content is delivered in the same order and at the same time across the intervention, and no additional texts would be sent from the content pool [66]. Therefore, based on the design features of the platform selected for the pilot study (see the Discussion section), it was decided that only 1-way text communication (mHealth to user) could be used for this iteration of the intervention. An automated feature of the sending technology is to cease the sending of any texts if a user sends a reply saying “Stop.”

## Discussion

### Principal Findings

This study describes the development of an mHealth intervention to support people with chronic pain who are tapering prescription opioid medications. Using a co-design methodology, we sought input from 12 consumers with chronic pain who had experience tapering prescription opioids and 12 clinicians with experience supporting opioid tapering in chronic pain treatment services to develop an mHealth program consisting of a 10-minute video and 56 mobile phone texts. On the basis of consumer perspectives (formative research [16]) and research and resources from the areas of digital health, chronic pain, and opioid tapering, content was developed in which consumers and clinicians agreed was, on average, acceptable, likely to be useful (consumer perspective), and likely to be effective (clinician perspective) in supporting consumers to taper opioids. Most study participants provided optional open-text feedback on the proposed script or the texts. Participant feedback on the proposed content enabled further revisions and improvements.

### Strengths of the Study

Consumers were involved in the conception and co-design of the intervention and intervention content. Consumers and experienced clinicians (“stakeholders”) were represented within the research team, and an independent group of consumers and clinicians participated in the evaluation of the initial content development and design concept. Stakeholder participation in co-design should improve consumer and clinician engagement with digital health interventions and may also help improve the retention of participants in subsequent pilot studies and trials of mHealth support [23-27].

The conceptualization of this mHealth support occurred before the COVID-19 pandemic having disruptive effects on human movement and increasing the need for and use of technology-assisted health care. The current content development research was conducted during a period where Australia, similar to many other countries, was engaged in a variety of social distancing measures and, at times, strict isolation and quarantine. Whether acceptability or preferred content delivery preferences changed within this timeline of disruption because of the COVID-19 pandemic remains to be determined. However, it could be predicted that as digital health technologies have become more normalized since the onset of the COVID-19 pandemic, the acceptability of mHealth interventions may have increased further since this study was conducted.

Various reviews have indicated the need for digital health interventions to support chronic pain [21,67,68]. Most of the interventions described in these reviews are web-based interventions and often include some degree of clinician support. A variety of digital interventions have been designed to support people with chronic pain, including texting interventions [69], the use of smartphone-based chatbots [70,71], and more sophisticated technologies such as virtual reality [72,73]. However, to our knowledge, no published studies have described mHealth interventions developed specifically to support people with chronic pain to reduce their reliance on opioids. Only 1 other planned research project has been identified, where the purpose is specifically to support individuals with chronic pain to reduce their reliance on opioids [74]. A pilot study protocol describes the proposed intervention, which uses a mobile app [74]. This study also used a co-design process for content development. The content developed in our study represents an advancement in the support available to individuals with chronic pain who are tapering, particularly end users who prefer to text over a more sophisticated app or other digital support [16,74].

### Limitations of the Study

#### Video Script

Notably, the participants did not evaluate the final video; they only read a script. This may have influenced the evaluation of the content, as video testimonials may be more engaging and persuasive than written testimonials [62]. Furthermore, despite the survey ratings indicating agreement on the appropriateness and likely effectiveness of the script to support consumers, only 7 clinicians provided text feedback for the script. To overcome these difficulties, the research group (including clinicians and a consumer representative) iteratively edited the video multiple times to ensure that it was of a professional standard.

#### SMS Text Messages

This study aimed to balance the desire for a rigorous co-design methodology with a need for efficiency and to reduce the demands on study participants. To address the latter issue, participants evaluated only 15 of the 90 texts from the draft list. We believe that the use of MSS and a cut-off of an MSS 8 to evaluate the content, which has been used in a similar study, was appropriate for our objectives [41]. However, because the data only included 2 participants’ ratings from each group per text to calculate an MSS, it is acknowledged that this is less representative than all participants rating all texts that we would have done if we believed it feasible for participants. In our study, 1 participant’s perspective significantly influenced the MSS. To mitigate this, open-text feedback was carefully considered, rather than relying solely on survey responses. Given the reasonably small sample groups (n=12 per group), it should be acknowledged that this study largely aimed to gain perspectives from individuals with experience in the domains of tapering and chronic pain using purposeful sampling and targeted recruitment of participants known to have chronic pain and experience tapering, or as a clinician with experience supporting this group. Therefore, the demographic information collected from participants, as reported in the paper, was minimal. The design could not be considered a completely representative sample of potential end users of the proposed mHealth support (Australians with chronic pain who are tapering prescription opioids). In retrospect, with the aim of describing the co-design methods in the development of this intervention and to facilitate evaluation and discussion in the academic community, demographic details should have been collected with more rigor. Despite this limitation, we believe that a good balance was struck to meet the aims of developing, pretesting, and revising the mHealth content minimizing the participant burden [28].

### Next Steps

An important step is to confirm and validate our mHealth content in a diverse group of patients (eg, age, race or ethnicity, and pain condition). A multisite pilot randomized controlled trial will be conducted to further evaluate the intervention using the content developed in this study [75,76]. The pilot randomized controlled trial will recruit consumers from pain clinics who are tapering opioids to evaluate the acceptability, feasibility, and preliminary effectiveness (improving tapering self-efficacy) of the mHealth content (video and texts) and usual care, compared with usual care alone [75,76]. Further revisions and refinement of the intervention (content and design) can be made based on feedback from the trial participants [28,76]. Of particular interest is the acceptability and feasibility of the videos. The video helps address consumer-identified needs for education about tapering and chronic pain before receiving texts [16]. However, the inclusion of internet-based videos (accessed through a website or downloaded) requires higher technological literacy and more sophisticated technology (eg, smart phones, tablets, or laptops with access to the internet) than receiving texts alone. Furthermore, while a range of strategies are used in this mHealth support, those included are not exhaustive, and various other strategies could be integrated into the text message format [46,77]. Feedback from trial participants may also identify potential modifications to the text. These could include manipulating delivery characteristics (eg, 2-way texting or changing the frequency of texts) [16] or content (eg, different information or communication strategies). Our chosen content development model encourages an ongoing process of piloting and reviewing content, and our future research will aim to continuously improve the content developed in this study [28]. A range of different consumer and stakeholder engagement strategies can be used in the codevelopment of interventions [78,79], and many commercially available chronic pain apps (mHealth interventions) do not feature co-design [80]. The model selected for the co-design in this study was chosen because it is specifically for text intervention development [28]. However, any researchers engaged in future research into mHealth co-design should explore various models and methods to ensure that their design methodology meets the needs of their end users and the systems that they will develop (eg, texting, app, or browser-based).

### Conclusions

Using a co-design methodology with 12 consumers and 12 clinicians, this study describes the development of a 28-day mHealth intervention consisting of a 10-minute video and 56 text messages. The intervention aimed to support people with chronic pain to taper prescription opioids. Overall, the content of the video and text messages were considered generally acceptable and likely to be useful and effective by both consumers and clinicians. Feedback provided by study participants informed improvements to both the script and texts, which should improve the acceptability of the intervention in a pilot randomized controlled trial. A randomized controlled trial will investigate the acceptability and feasibility of the mHealth intervention among a group of patients with chronic pain recruited from tertiary pain clinics who are tapering prescription opioids under the supervision of a pain specialist [75,76].

## Appendix

Multimedia Appendix 1 Summary of open-text feedback for script and text messages, final script, examples of the review process for text messages, and text message content.

## Abbreviations

mHealth: mobile health
MSS: mean sum score
REDCap: Research Electronic Data Capture
