# Supplementary material for: A Digital Video and Text Messaging Intervention to Support People With Chronic Pain During Opioid Tapering: Content Development Using Co-design
Source: JMIR Form Res. 2022 Nov 10;6(11):e40507. doi: 10.2196/40507 (PMC9693745; doi:10.2196/40507)
Supplement: Multimedia Appendix 1 [file formative_v6i11e40507_app1.docx]

## Supplementary File: A DIGITAL VIDEO AND TEXT MESSAGING INTERVENTION TO SUPPORT PEOPLE WITH CHRONIC PAIN DURING OPIOID TAPERING: CONTENT DEVELOPMENT USING CO-DESIGN

## Open Text Feedback for Script and Text Messages

Script- Consumers
General Comments

- Sometimes as much as you try to "break through" the pain it becomes stressful and it effects your mental state of health (Consumer 1)
- Further, there seems to be emphasis on stress reduction, which although I totally agree with, I would argue this is a barrier for some patients. In patients privy to emotionally-led decision making, a stressful or unexpected event may derail the entire opioid-reduction process. In such a case, there may be a consequential hesitancy to begin. (Consumer 9).

#### Positive Feedback, No Suggestions

- With the knowledge it's a video script, I think it's an appropriate and wholistic start to the program. (Consumer 9)
- The introduction to elements like mindfulness, nutrition, exercise etc. gives a full snapshot. (Consumer 9)
- I think that it is very useful using Patient examples in the video. Everyone experiences pain differently, so seeing someone that has gone through something similar will be beneficial. (Consumer 12)

#### Positive Feedback, Suggestions

- I appreciate the push for setting up your support network at the end, with the assumption most pain patients have this available (think rural/isolated patients who may not). I would add in the support network paragraphs, a reference to preparing for yourself; how can you make a potentially tricky period of time easier for yourself? Things like preparing your meals ahead for days where all you may otherwise feel like doing is getting takeaway - a terrible option if trying to keep your pain to a minimum. Adjusting expectations at work; time-off or task adjustment may be called for. In all, preparing ahead to give yourself the best chance at success. This may be better put in later modules. (Consumer 9)
- The statements from Patients 1, 2, & 3 will be useful for some, however there are a few key factors which really do need emphasing, and reinforcing: You must have a very strong desire to be successful at achieving the goal, I draw parallels to a person who wants to stop smoking. No matter how many nic patches, nic gum, phone calls you make etc, if you don't want to do it, then you're wasting (everybody's) time attempting to do it. [emphasise] The real health benefits of being off all pain meds, not just the physical (there's a real & tangible progression), The feeling of "accomplishment/success" when you achieve your goal. (Consumer 11)
- My immediate thoughts were that it does encapsulate everything you think patients may want/need to hear, however I must admit that it does confuse me to some degree.

If its to be viewed by a patient that is a long term pain med user, new to the idea of reducing medications, and this is their first real exposure to what lies ahead, then I feel that there needs to be tweaked a little. For example, one of my biggest mental obstacles was understanding the difference between Acute Pain and Chronic Pain. It wasn't until [Clinical Psychologist] thoroughly explained the differences (in particular, the fact that chronic pain was something I would always have), could I begin to work on the mental aspect. This would be a crucial factor in my ability to move forward. (Consumer 11)

#### **N**egative Feedback, No Suggestions

- A little to basic. (Consumer 2)
- Limited coping mechanisms provided. (Consumer 2)
- Looking back when I was in "real" pain the breathing exercise etc...did not help...what helped was the medication. I tried hard to breath, to distract myself but it simply hurt to much to do this...so when I read about it it makes me a bit sceptical (Consumer 3)
- Patient 1, first section careful ref pacing, or bring it into subject about a balance when doing everything…Patient 3 in first section commencing "my pain now is xxxx" needs rewording, makes no sense...Patent 2 in second section reword about family…P 3 second section complete the sentence or 'You need to have -' is it a shrug ? (Consumer 5)
- I would suggest a greater look into pacing might help in this script. I see the reference to "Planning your day," however it is bundled in with a reference to nutrition. Pacing was a key concept that assisted me in my reduction was when I hit walls whilst reducing, the medical team helped me to access how to pace down activities so I could complete them. (Consumer 9)

#### Negative Feedback, Suggestions

- I found the paragraph about responding to SMS messages confusing. Would my answers as to whether I wanted more, or fewer SMS messages be classed as general or specific messages? Perhaps unnecessary to try and explain in such detail about this. It was a negative administrative note to finish on after an encouraging tone to rest of presentation. (Consumer 4)
- The patient examples come across as "already recovered" and would therefore be potentially not relatable to people still struggling with chronic pain management, and were solution-centric without describing adequately the steps taken so far to achieve their success. A success story with a clear description of how they managed to achieve those results over time would be much more helpful. And the information that there are multiple possible pathways to success is also important to cover. (Consumer 10)

#### Suggestions without feedback

- Change 1 in 5 to 20% (Consumer 5)
- List triggers for taking action on your pain. For when it might not be chronic. (Consumer 8)
- My pain meds intake had been steadily increasing for years, and it was always caveated by "its so you can have a better standard of life". So perhaps a little bit more information pertaining to this may be useful? (Consumer 11)

### Script- Clinicians

#### General Comments

- Is this a video participants can watch multiple times/show family etc? (Clinician 1)

#### Positive Feedback, No Suggestions

- Are they real patients? or actors? The cases can be very helpful. (Clinician 1)
- Repetition re. values based activities is very good. (Clinician 2)
- it's good that the language is simple and people can relate to the examples used. (Clinician 5)
- Overall, a good script and obviously alot of thought has gone into the lay examples and terms. (Clinician 6)

#### Positive Feedback, Suggestions

#### Negative Feedback, No Suggestions

- The reason I was neutral is the I have found the effectiveness of ‘information giving' depends very much on the patient's motivation to change/engage. I'm not sure how effective it will be if people are very pre-contemplative about dose reduction. (Clinician 1)
- The script is long. It takes many many paragraphs to get to the point that the study is about supporting opioid reduction because opioids don't "work" for chronic pain. (Clinician 10)
- Opioids (in the context of chronic pain) are unhelpful- they create dependency and reduce people’s ability to care well for themselves. This is not really highlighted. (Clinician 10)
- There is no 'apology' for the dilemma patients find themselves caught up in. It presumes patients do not already "understand" their pain. (Clinician 10)
- It often refers to "our" pain or "we". In my view it is "your pain" and "you" (Clinician 10)
- It does seem that the script is promoting 'self-management" of pain / and targeting the "nervous system" is the goal, rather than reduction of pain via actively treating the pain system wholistically. Do patients know what a nervous system is? (Clinician 10)
- It does at one point highlight distracting from pain as a good strategy// I would tend to disagree- wea sk patients to tune in to pain and develop a broader awareness of what is going on internally (Clinician 10)

#### Negative Feedback, Suggestions

- In 2021, I would hope that very few patients and clinicians would be able to relate to this in the management of non-malignant pain, so can we make it more realistic: [Patient 3] "In basically just over 2 years, I've gone from 240mg of oxycontin to 30mg of oxycontin. Maybe Targin 40/20mg twice a day to10/5mg twice a day? (Clinician 6)
- A lot of information is presented. I wonder if less information and more recognition of potential emotional distress might be helpful. Perhaps also greater acknowledgement of the bravery of the person in beginning or considering the opioid reduction path. (Clinician 8)

#### Suggestions without feedback

- Personally I find people relate more to a faulty smoke alarm rather than a car alarm. Most people have experience with a smoke alarm going off when they burn to toast, or just boil kettle. But car alarm works too. (Clinician 1)
- Only other comment is that perhaps use some language like 'reducing your dose' rather than just 'tapering', which is a higher literacy level word. (Clinician 1)
- "drinking to excess" - could actually name ETOH (alcohol) to be more clear. (Clinician 2)
- Suggest to include cultural, gender and age inclusivity etc when selecting actors for the script. (Clinician 2)
- Perhaps when talking about flare-ups, mention of the role of stress. (Clinician 3)
- Strategies to help with relaxation could include stretches - assist in raising awareness of increased muscle tension and the learning to let go of the muscle tension using the out breath. (Clinician 3)
- Could have greater emphasis on self management strategies to assist with tapering. (Clinician 3)
- I wonder too about making specific mention of the role of the GP (often the prescriber) and the need for close coordination between GP and specialist team. (Clinician 8)
- How about addressing the amount of influence that the patient might or might not have over timing of when to start reducing and rate of reduction. Letting the person know that they will have a voice can allay anxiety to some extent. (Clinician 8)

### Text Messages (General Feedback, not item level)- Consumers

#### General Comments

- A few things that went a long way towards helping me get off the meds: Setting myself monthly goals (reducing drugs "x" amount per month etc) in consultation with GP…I used the Webster Packs: my pharmacy would prepare 4 weeks at at time (very very helpful)… establishing a daily routine: time to get up, exercise/gym, eating, bed time, sleeping etc…(I would strongly encourage this type of behaviour - it helps eliminate time focussed on pain)…The meditation techniques taught by [Clinical Psychologist] instrumental in overcoming the pain and negativity. I was a sceptic - but only once :)…Regular meetings with your pain management team…Simply being able to speak to people who genuinely understood what chronic pain was, and had solutions, and systems in place to support you, was essential and vital to my success. (Consumer 11)
- Above all was my desire to feel & be, in control of my life. I wanted to be able to think clearly, go places, and do things without the dependence of drugs. I wanted to feel and be normal. (Consumer 11)

#### Positive Feedback, No Suggestions

- My above responses have been additional helpful comments I would have appreciated hearing earlier on in my own journey many people struggling to manage their pain need education and options, the more information they can access the better. (Consumer 10)

#### Positive Feedback, Suggestions

- Every day is great. Please do not write tomorrow will be better... (Consumer 3)

#### Negative Feedback, No Suggestions

- But i only got through what was happening to me because the 'why' was explain, not just the highlights and it gets told to you generically. These are generic statements, understanding the why in plain English is needed (Consumer 8)

#### Negative Feedback, Suggestions

#### Suggestions without feedback

- Hi, I think these inspirational messages should be sent 1st thing in the morning for motivation and in the afternoon when we start lagging and each day (Consumer 1)
- Message time to be later in the day, say late morning. (Consumer 2)
- Support is so important…Empathy to what the person is feeling there and then is important…Validate how hard it is...this is very important. (Consumer 3)
- Morning message is very important as usually after a night of not taking the opioid the withdrawal is intense. Another one in the afternoon (4ish/5ish) another one to validate the day... (Consumer 3)
- Wondering whether the clients could initiate the receipt of a text when they feel they need a boost rather than have one sent to them. I'm picturing myself feeling irritated by an upbeat text arriving unsolicited when I'm already feeling grumpy but grateful for that same text if I seek out some support myself. Just a thought. (Consumer 4)
- Evenings & first thing for many of them - I've noted some that I feel need to be timed for different scheduling during the tapering process (Consumer 5)
- Keeping the messages short and sweet is good. (Consumer 8)
- Change the language to a more active voice rather than passive. It will draw the audience in and help them to check in with themselves. (Consumer 9)
- As for timing of the messages, I can only say what I would have found useful for me... the first one in the morning: around 7.30-8.00am. This should be one offering encouragement etc for the day ahead. A second definitely in the evening: 12 hours later. Congratulating you getting through the day and/or words to assist in preparing for bed/sleep (eg) "meditation time" (not "medication time" nurse ratchet) (Consumer 11)
- I think that the time of day the messages are sent should be individualized. For example, I work night shift, so having a message sent at say 9am when I am asleep wouldn't be useful for me. The participants should be able to choose what time will work best for them (Consumer 12)

### Text Messages (General Feedback, not item level)- Clinicians

#### General Comments

#### Positive Feedback, No Suggestions

- I agree with the proposed content of the messages. Good that the frequency is individualised. (Clinician 3)

#### Positive Feedback, Suggestions

- It’s great. I've written suggestions in the other boxes. I think perhaps shorter, cleaner texts would be my main suggestion (Clinician 10)

Negative Feedback, No Suggestions
Negative Feedback, Suggestions

- I prefer the language that is less instructive, more informative. eg instead of 'stay hydrated and drink plenty of water', I would be more neutral eg - 'staying hydrated and drinking water should reduce withdrawal symptoms'. Less bossy... (Clinician 1)

#### Suggestions without feedback

- Sending variable times and days will increase any potential dopamine release and improve engagement, ie. make it unpredictable (Clinician 2)
- maybe look to make some of the messages more specific to certain things and add some context perhaps? (Clinician 5)
- 15 messages so maybe 2 SMS a day so all sent are over a week sending 1 in the morning around 8am and a 2nd around 5pm each day (Clinician 6)
- I think two per day, one to start the day that has some strategies eg relaxation and one at the end of the day/late arvo that is more reassuring eg tomorrow is a new day/keep at it etc. (Clinician 11)

Final Script

**Narrator:**You are watching this video today because your doctor has recommended you reduce your opioid medication dose. This can be a daunting prospect and many people find that it helps to have information and support. This video presents a few strategies for managing your pain and maintaining your health and well-being as you reduce your dose of prescription opioid medications.

What helps? Understanding the meaning of your pain helps.

Knowing that chronic pain is more about sensitivity than damage can help reduce the worry that often goes hand in hand with the experience of chronic pain. Chronic pain is a bit like an over-responsive smoke alarm that goes off when you are cooking or having a steaming hot shower. The loudness of the alarm is real and distressing but there's no fire or real danger that we need to protect ourselves from.

The less that we worry about our pain, the better our quality of life. Pain flare-ups can happen from time to time, especially when we overdo things or overstimulate our nervous system. Planning your day to include time out, setting realistic goals for the day, eating healthy food, and avoiding caffeine and alcohol can all help to manage pain flare-ups.

Research has shown that doing things that help you to relax can have an immediate effect on your pain. Relaxation strategies can be especially useful for reducing the intensity or unpleasantness of pain when we are feeling overwhelmed. What helps you to relax? There are many things that people tell us help them to relax and in turn manage their pain. These include deep breathing, muscle relaxation, meditation, reading, gardening, chatting with a friend, getting some fresh air, and spending time in nature. It's important to find things that work for you. Talk to your healthcare provider for further ideas on how you can relax your mind and body.

Sleep also helps. It's normal to have trouble sleeping when you have pain, but sleep is important to help your mood and maintain energy levels. Some things that help with sleep are Reducing screen time before bed, maintaining a regular sleep routine, avoiding alcohol and caffeine, and trying to keep active during the day.

Often when we are distressed by pain it can deter us from participating in activities that are important to us. Many people reduce their physical activity in response to pain, but gentle movement is one of the best remedies for pain. Physical activity is good for your mind, your mood, and your energy levels.

You may be wondering why opioid medications are no longer recommended for daily pain management. Opioid medication is effective in reducing some types of pain, like pain after surgery. However, evidence shows these medications are not very useful in helping to manage chronic pain. This is because the pain-relieving effects of opioids decrease over time.

Some people feel that taking increasingly greater doses might be helpful, but this increases the unpleasant side effects of these medications like constipation, nausea, drowsiness, sexual dysfunction, low energy, reduced concentration, depression, and if that's not bad enough, in some cases long-term use of opioids can even cause more pain. This can happen because when people take opioid medication the body's natural pain relief systems become disrupted and more sensitive and this can make pain worse than before.

When people gradually reduce their opioid dose over time, with the support of a healthcare professional, many people find that they can manage pain just as well, if not better, than when they were taking opioids. Some people also report having better concentration,

improved mood, more energy for activities, improved confidence to manage their pain without medication, better relationships, and improved day-to-day functioning. This is especially true if people use pain self-management strategies such as relaxation, staying active, and getting a good night sleep.

Many people report feeling concerned that their pain will get worse when they are reducing their opioid dose. It's true that people often experience fluctuations in pain while reducing their dose, but it doesn't necessarily get worse. Pain can change from day to day, but over time people generally find it gets better as they reduce their medication. There are many strategies that people find helpful for managing pain flare-ups such as relaxation, gentle movement, and stretching. Try different strategies. You have to find what works for you. If you have trouble, contact your healthcare provider for additional support.

Some people do experience symptoms of withdrawal. While these symptoms can be unpleasant it’s important to remember that they are temporary. In fact, many individuals with chronic pain experience little or no withdrawal symptoms while reducing their opioid dose because they reduce the dose very gradually. Everyone's experience is different, but here's a few individuals telling their stories about reducing their opioid medication dose. We've asked them to tell us about their experience and what worked for them.

**Person 1:**
“I've felt so much better to be off the tablets, absolutely wonderful. I've felt stronger, and after that I've learned to deal with the pain itself without the painkillers. I didn't feel I had a life, I felt very enclosed, low, constantly tired, just I didn't want to socialize because I was so tired and rundown and in pain. Once you know what a flare-up is and how to deal with it, it becomes a lot less scary and severe and you just go 'oh yeah I'm having a flare up, all right, let's deal with this'.”

**Person 2:**
“I used to be on the pain medication for three years. That's a lot for me, and I can tell you that it's actually kind of stopping me from enjoying my life a lot. It's not easy but if you try you will see how much more you can enjoy your life and I actually found that, that the pain medication is not everything. So now I got pain, but I enjoy my life a lot. I go shopping, I go outside, I go do a lot of outdoor activity stuff with my friend, with my partner, and I love them.”

**Person 3:**

“The pain is there. I mean I've got terrible back pain, but the way I manage it is first thing I work, and I love my work, so I work about eight hours a day, in pain, but it distracts me. Exercise is the key to everything I believe. I try to exercise nearly every day. Even yesterday I exercised, and my back was killing me, and I just kept on saying to myself get over it and it's not easy to say that but do what you can do. Don't think of what you can't do.”

**Person 4:**

“In basically just over two years I've gone from 240 milligrams of oxycontin to 30 milligrams of oxycontin. I am feeling really proud of myself, and I have to remember that at times also I've done it with a lot of help, but I’ve done it. I'm more social, I'm doing more, I'm enjoying life a hell of a lot more. You need to have [support] you can’t do it all on your own, without a doubt. Even these small amounts to begin with. And involve maybe as many people as you

want or are comfortable with, but you do need, I say to everybody, supportive people around you.”

**Narrator:**As you've just heard from people with personal experience, it helps to have support around you when you start to reduce your opioid medications.  Reach out to your friends and family for support and continue to seek information and guidance from your healthcare provider.

In addition to this, we will support you by sending you a couple of text messages a day

over the next four weeks to provide you with information and strategies that have been helpful for others.

Each text message has been written together with patients who have experience reducing opioid pain medications and they have been also endorsed by clinical experts. Researchers have found that receiving daily text messages can really help people to change their behaviour and habits. We look forward to hearing about your experiences.

## Figure 1. Examples of the review process for text messages

Consumer MSS=9

Consumer feedback:

“*Link with doing relaxation with somebody if really tense or use a tape that is specific to you.*”

Clinician MSS = 9.5
No clinician feedback

Revised message, included in final list

“Relaxation strategies can help to reduce muscle tension and pain. Try deep breathing exercise for a few minutes several times per day. Finding a recording online that works for you can be a great help too!”

Message 7, pain education and self-management:

“Relaxation strategies can help to reduce muscle tension and pain. Try deep breathing exercise for a few minutes several times per day.”

Consumer MSS=7.5

Consumer feedback:

*It is important to remember that if you have had experience with long term opioid use, even short term use will need to be closely monitored as it can open you to thinking you may need them long term again.*

Clinician MSS = 6
Clinician feedback:
*It seems like a backward step to receive a message promoting opioids for acute pain and irrelevant.*

Removed message from library.

Accept feedback from clinician reviewer.
Unnecessary message.

Message 35, opioid tapering support

“Opioid medications can be useful for pain in the short term (a few days or weeks). However, research shows that opioids offer only limited relief in the long term.”

Consumer MSS=9
Consumer feedback:

*“Soften the first sentence. It comes across as a barking order. [trt] ‘Have you been pacing yourself? Take regular breaks and be [...] in activity. This short term pain is for long-term gain’ "
“Pacing can be incredibly challenging and takes practice to master. Start small, focus on gradual increases in the activity you are aiming to pace. Give your body time to adjust before increasing. Be specific about what activities it is important to pace; even house chores and daily activities can impact energy levels and pain, therefore require pacing.”*

Clinician MSS = 7.5
Clinician feedback:
*“Lots of messages in here. Pacing is usually avoidant behaviour in my view. I would say, get support to gradually increase your activity levels”*

Revised message, included in final list

“Have you been pacing yourself? Take regular breaks and be get support to gradually increase your activity levels. This short term pain is for long-term gain.”

Message 15, pain education and self-management:

“Pace yourself. Take regular breaks, be mindful how your body copes with gradual increases in activity, and look for long term gain not short term pain”

Consumer MSS=8.5

Consumer feedback:

*“I would have expected this information to be already known. GP's etc are obligated to advise a patient accordingly, prior to administrating the drug/s - y/n?”*

Clinician MSS = 8
No clinician feedback

Did not revise message, included in final list

Response to feedback: This message is not new information but is a statement of fact. It’s important to reinforce the down sides of taking opioids

Message 43, opioid tapering support:

“Opioid medications have many undesirable side effects including constipation, drowsiness, fatigue, dry mouth, and nausea. There is also a risk of dependence and overdose.”

Consumer MSS=8

No consumer feedback:

Clinician MSS = 9.5
No clinician feedback

Message revised to reduce reading demands. Included in final library.

After reducing your opioid dose, you may find you can get more out of each day.

Message 59, motivation to taper opioids:

“Many people with chronic pain experience improved functioning after tapering opioids. They are better able to do tasks and feel more in control at home and at work.”

Consumer MSS=8

No consumer feedback:

Clinician MSS = 9.5
No clinician feedback

Message revised to reduce reading demands. Included in final library.

“After reducing your opioid dose, you may find you can get more out of each day.”

Message 59, motivation to taper opioids:

“Many people with chronic pain experience improved functioning after tapering opioids. They are better able to do tasks and feel more in control at home and at work.”

Consumer MSS=8

Consumer feedback:
*“Just don't add*

*Tomorrow is a*

*new day.”*

Clinician MSS = 9.5
No clinician feedback

Message revised in line with feedback. Included in final library

“Ups and downs are to be expected. If you are feeling down or having a bad day, cut yourself some slack.”

Message 77, motivation to taper opioids:

“Ups and downs are to be expected. If you are feeling down or having a bad day, cut yourself some slack. Tomorrow is a new day.”

Consumer MSS=8.5

Consumer feedback:
*“Try; "Mood changes? this can be a symptom of Opioid withdrawal" it engages the audience more.*”
*“Methods for mood tracking or ongoing support with emotional management can be beneficial to recovery.”*

Clinician MSS = 8.5
Clinician feedback:
The initial video did not point out that people may be using opioids as a means of regulating their anxiety, therefore withdrawal of them is likely to lead to some disregulation.

Message revised in line with feedback. Included in final library

“Mood changes? Changes in your mood can be a symptom of opioid withdrawal. While this can be unpleasant, withdrawal symptoms are temporary, and will reduce with time.”

Message 80, motivation to taper opioids:

“Changes in your mood can be a symptom of opioid withdrawal. While this can be unpleasant, withdrawal symptoms are temporary, and will reduce with time.”

## mHealth Text Message Content

| **Day 1-28** | **AM or PM** | **Final Message** |
| --- | --- | --- |
| **1** | **AM** | Welcome to our study! You will receive 2 text messages per day from us over the next 4 weeks. Each message is designed to help support you during your opioid medication reduction. Save this phone number as TXTSupport@USYD. Text STOP if you want to opt out of the study. |
| 1 | PM | Hi #firstname#, There are many ways to manage pain without opioids and lots of resources to help with pain self-management are available online. Here's a good place to start: https://aci.health.nsw.gov.au/chronic-pain. USYD |
| 2 | AM | Hi again #firstname#. When people reduce their opioids, they are able to think more clearly and do more to look after themselves and others. USYD |
| 2 | PM | By taking fewer opioids, many people find they are able to think more clearly, be more active, and participate in more social activities. Less pills, more life. USYD |
| 3 | AM | Withdrawal symptoms can be avoided or minimised when you taper slowly and look after your health. Good nutrition, sleep, and exercise will help. Introduce small sustainable changes over time. USYD |
| 3 | PM | Pain flare ups are like bad moods. Sometimes they come on for no clear reason. They will often pass if you distract yourself. Try doing or thinking about something you enjoy. USYD |
| 4 | AM | For many people, less opioids means feeling safer driving, being better able to engage in daily activities, experiencing better sleep, and better mood. USYD |
| 4 | PM | Hi #firstname#, withdrawal symptoms while reducing your opioid medication are temporary and will pass. USYD |
| 5 | AM | There can be many benefits to reducing opioid medication. A lot of people with chronic pain find their mood and energy levels improve when they taper their opioids. USYD |
| 5 | PM | Relaxation strategies can help to reduce muscle tension and pain. Try deep breathing exercise for a few minutes several times per day. Finding a recording online that works for you can be a great help too! USYD |
| 6 | AM | Hi again #firstname#. Drinking plenty of water can help with withdrawal symptoms such as sweating and stomach upset. USYD |
| 6 | PM | Don’t feel ashamed if you’re finding it hard. You’re not alone. Many people with chronic pain experience challenges while tapering. However, most of them say it was worth it in the end. USYD |
| 7 | AM | #firstname#, when you do things that engage you fully and make you lose track of time, you may lose track of your pain. USYD |
| 7 | PM | It's normal if you feel irritable or low while reducing opioid doses. It's a big change and your body needs time to adjust. Try to be kind to yourself. USYD |
| 8 | AM | Good morning #firstname#. Try not to take on big challenges while reducing your opioid medications. You don’t want to make things more stressful for yourself when you are already making this big change. USYD |
| 8 | PM | After reducing you'll be able to think more clearly, your sleep will improve, and you’ll be less constipated. USYD |
| 9 | AM | Stress can increase pain, and pain can increase stress. Stress management strategies like relaxation or light exercise can help to manage pain. USYD |
| 9 | PM | There is no “normal” when it comes to withdrawal symptoms while reducing opioids. If you experience them they typically subside in less than a week. USYD |
| 10 | AM | #firstname#, you are doing your best in a challenging situation. Be proud of yourself. USYD |
| 10 | PM | Opioids can make chronic pain worse over time. Many people find that their pain improves after a period of time off opioids. USYD |
| 11 | AM | Pain can change based on things like stress, nutrition, movement, sleep and your mood. Changing these things can help to reduce pain. USYD |
| 11 | PM | Here are some tips to improve sleep. Try getting up and going to bed at the same time each day, avoid long naps, increase activity, reduce screen time before bed, only use your bed for sleep, and reduce caffeine and alcohol. USYD |
| 12 | AM | Consider your relaxation strategies as medicine that you use regularly. Setting the alarm on your phone to remind you to practice deep breathing, stretching, or walking a few times a day. USYD |
| 12 | PM | Having mood changes #firstname#? Changes in your mood can be a symptom of opioid withdrawal. While this can be unpleasant, withdrawal symptoms are temporary, and will reduce with time. USYD |
| 13 | AM | Many people with chronic pain report feeling healthier and happier when they take less opioid medication. Fewer pills, fewer side effects. USYD |
| 13 | PM | Reducing your opioid dose can be hard in the short term, but you will be much better off in the long term. Keep at it! USYD |
| 14 | AM | Plans give you something to look forward to. Plan what you'd like to do or achieve each day or week. And be kind to yourself if things don’t go to plan #firstname#. USYD |
| 14 | PM | Managing your pain, medications, withdrawal symptoms, and emotions can feel like a full-time job. Be sure to get the support you need and take your time. USYD |
| 15 | AM | Focus on self-care. Make sure you are getting enough rest. Eat healthy foods. Move regularly and get fresh air. Stay in touch with your friends. Try at least one of these forms of self-care today. USYD |
| 15 | PM | Hi #firstname#, you might be finding it hard to do the things you normally do due to withdrawal symptoms like mood swings or fatigue. As your body adjusts your wellbeing and functioning will improve. USYD |
| 16 | AM | One of the main reasons many people with chronic pain decide to reduce their opioid medication is because of the side-effects of long-term opioid therapy. As you cut down many side-effects pass quickly but some take longer. Your body will readjust. USYD |
| 16 | PM | Some days will be difficult. That's to be expected. Be proud of yourself for getting this far #firstname#. You are stronger than you realise. USYD |
| 17 | PM | Negative thoughts increase stress and stress can increase pain Thoughts are just thoughts. Thoughts are not always true or helpful. Taking a step back to your thinking or doing something different helps stress. USYD |
| 17 | PM | Some withdrawal symptoms pass quickly, others take more time. In all cases, withdrawal symptoms will pass. Remember there is light at the end of the tunnel. USYD |
| 18 | AM | Good morning #firstname#. After reducing opioid medications, you may start to find you are getting more out of each day. USYD |
| 18 | PM | Deep breathing can help manage stress and pain. Breathe in deeply for about 4 counts - feel your stomach expand. Hold that breath for 2 counts and then breath out slowly. Notice the relaxation spread through your body. Try this for a few minutes. USYD |
| 19 | AM | The support of family and friends throughout this can be helpful. Perhaps give one of them a call today to let them know how you are doing. USYD |
| 19 | PM | Opioid medications have many undesirable side effects including constipation, drowsiness, fatigue, dry mouth, and nausea. There is also a risk of dependence and overdose. USYD |
| 20 | AM | It can be tempting to try to get everything done on your good days. It can be hard to maintain your routine on bad days. #firstname#, try to be consistent with activity on good and bad days. USYD |
| 20 | PM | Opioid medication becomes less effective over time. After a gradual reduction, many people report their pain is more manageable and less distressing. USYD |
| 21 | AM | Most people take pain medications to relieve pain so they can do more. But in the long term, pain medications can increase pain which limits activity levels. USYD |
| 21 | PM | Practice talking to yourself as you would speak to a friend. Allow yourself the same compassion and understanding that you would offer to someone else. USYD |
| 22 | AM | Hi #firstname#, Exercise can improve your mood. Sometimes it’s hard to exercise if you are feeling down or in pain. But getting active – like going for a walk – can really help. USYD |
| 22 | PM | Hi again #firstname#, many people find their mood improves after cutting down on opioid medications. Keep that in mind throughout this process, particularly if you're having a bad day. USYD |
| 23 | AM | People often worry about increased pain when they taper off opioids. However, many people report less pain once they have cut down their dose. USYD |
| 23 | PM | It's normal to worry about reducing your medication. Take one day at a time. Just do the best you can today. USYD |
| 24 | AM | Motion is lotion! Gradually increasing the amount of activity you do each day is one of the most tried and tested ways of managing pain. USYD |
| 24 | PM | Long term opioid use can reduce our ability to experience joy and delight. Especially with high doses. Living with pain is no fun as it is. Adding the long-term side effects of opioid use can make things even worse. USYD |
| 25 | AM | Being social is important for your health and wellbeing and can help with pain. Reach out to friends and family, even if it’s just a brief phone call. You might be surprised how much it helps. USYD |
| 25 | PM | Some days the idea of tapering will seem more difficult. Other days, your pain will be better and more manageable. Hang in there! USYD |
| 26 | AM | Lowering opioid use and consistently using active strategies like regular exercise, pain education, and relaxation can improve physical, emotional and social well-being. USYD |
| 26 | PM | Hello #firstname#, ups and downs are to be expected. If you are feeling down or having a bad day, cut yourself some slack. USYD |
| 27 | AM | Maintaining a goal-focused and optimistic mindset helps with setbacks. Going through opioid dose changes can have ups and downs. Remind yourself of difficulties you’ve overcome before and that you can get through this. USYD |
| 27 | PM | If you’re having a bad day with pain or withdrawal symptoms, try some activities that you “get lost in”. This might be gardening, reading, walking, helping others, or listening to music. Be sure to still keep to your limits whilst you enjoy yourself! USYD |
| 28 | AM | Pain fluctuates over time but this is not a sign that pain will be worse in the long term. Indeed, research shows many people experience an overall unchanged or even less pain after reducing opioid medication. USYD |
| 28 | PM | #firstname#, pat yourself on the back. It is a real achievement to cut down opioid medications. Thank you for participating in the trial. This is the last message you will receive from us. You will receive a feedback survey shortly. We hope you found this information useful. USYD |
